# The Quandary of DNA-Based Treatment Assessment in De Novo Metastatic Prostate Cancer in the Era of Precision Oncology

**DOI:** 10.3390/jpm11050330

**Published:** 2021-04-22

**Authors:** Sigve Nakken, Wolfgang Lilleby, Marta D. Switlyk, Karen E. Knudsen, Oscar Lilleby, Sen Zhao, Fatemeh Kaveh, Per O. Ekstrøm, Alfonso Urbanucci, Eivind Hovig

**Affiliations:** 1 Department of Tumor Biology, Institute for Cancer Research, Oslo University Hospital-Radium Hospital, 0424 Oslo, Norway; sigven@ifi.uio.no (S.N.); senz@ifi.uio.no (S.Z.); fatemeh.kaveh@rr-research.no (F.K.); Per.Olaf.Ekstrom@rr-research.no (P.O.E.); alfonsou@ifi.uio.no (A.U.); 2 Centre for Cancer Cell Reprogramming, Institute of Clinical Medicine, Faculty of Medicine, University of Oslo, 0316 Oslo, Norway; 3 Department of Oncology, Oslo University Hospital-Radium Hospital, 0424 Oslo, Norway; WLL@ous-hf.no; 4 Department of Radiology, Oslo University Hospital-Radium Hospital, 0424 Oslo, Norway; marswi@ous-hf.no; 5 Department of Cancer Biology, Thomas Jefferson University, Philadelphia, PA 19107, USA; Karen.Knudsen@jefferson.edu; 6 Faculty of Health and Medical Sciences, University of Copenhagen, 2200 Copenhagen N, Denmark; oscar.lilleby@outlook.com; 7 Centre for Bioinformatics, Department of Informatics, University of Oslo, 0315 Oslo, Norway

**Keywords:** prostate cancer, disease management, genomic testing, guidelines, treatment biomarkers

## Abstract

Guidelines for genetic testing have been established for multiple tumor types, frequently indicating the most confident molecularly targeted treatment options. However, considering the often-complex presentation of individual cancer patients, in addition to the combinatorial complexity and inherent uncertainties of molecular findings, deriving optimal treatment strategies frequently becomes very challenging. Here, we report a comprehensive analysis of a 68-year-old male with metastatic prostate cancer, encompassing pathology and MRI findings, transcriptomic results, and key genomics findings from whole-exome sequencing, both somatic aberrations and germline variants. We identify multiple somatic aberrations that are known to be enriched in prostate cancer, including a deletion of PTEN and a fusion transcript involving BRCA2. The gene expression patterns in the tumor biopsy were also strikingly similar to prostate tumor samples from TCGA. Furthermore, we detected multiple lines of evidence for homologous recombination repair deficiency (HRD), including a dominant contribution by mutational signature SBS3, which is specifically attributed to HRD. On the basis of the genomic and transcriptomic findings, and in light of the clinical case presentation, we discussed the personalized treatment options that exist for this patient and the various challenges that one faces in the process of translating high-throughput sequencing data towards treatment regimens.

## 1. Introduction

The concept of precision oncology, defined by the American Society of Clinical Oncology (ASCO) as “the molecular profiling of tumors to identify targetable alterations”, has been widely embraced by nearly all clinical disciplines. Next-generation sequencing (NGS) is widely acknowledged as a critical technology to realize the full potential of precision medicine in clinical practice [1,2]. Nonetheless, from a clinical point of view, the determination of an optimal treatment strategy remains a quandary in the backdrop of the often complex, clinical reality of a case. The multitude of potential combinations of alternative treatment strategies, yet to be fully validated by robust research, adds to this complexity. Here, we present a prostate cancer (PCa) case diagnosed with metastatic disease at first presentation that illustrates some of the many challenges arising from the availability and use of novel treatment modalities.

In 2015, the CHAARTED study showed that the combination of docetaxel and castration increased the overall survival in men with primary metastatic prostate cancer (mPCa) by 22 months, compared to standard therapy with androgen deprivation therapy (ADT) alone [3]. In 2018, the STAMPEDE trial reported a survival benefit of 8% (HR 0.68, 3-year OS 73% vs. 81%) in a per protocol predefined subpopulation of men with low-tumor volume PCa adding local radiation to the prostate [4]. However, the clinical prospects in men with mPCa remain poor, eventually leading to resistance development and fatal outcomes.

An increasing emphasis on the concept of precision oncology has led to a paradigm shift moving away from Virchow’s “omnis cellula e cellula” cell biology-centric approach to a more granular focus on genomic, transcriptomic, and proteomic alterations observed in cancer cells and the surrounding tumor microenvironment. Our understanding of PCa at the molecular level has been significantly improved by several large-scale sequencing efforts, including The Cancer Genome Atlas (TCGA), which has characterized approximately 500 primary prostate tumor samples at the genomic and transcriptomic levels [5,6,7,8].

Despite the limited clinical implementation at present, the number of PCa patients potentially benefiting from NGS-based molecular profiling has been reported to be in the order of 30%. This estimate is defined by the set of cases harboring loss-of-function variants (somatic or germline) in DNA repair genes, which, in turn, indicates that these tumors may be sensitive to PARP inhibition (PARPi) or an immune checkpoint blockade [6]. Not least, the particular findings of genomic alterations in genes involved in DNA damage repair, combined with the efficacy of PARPi targeting the DNA repair machinery, establishes the evolving importance of this strategy in the armamentarium for treating mPCa [9]. The foregone changes highlight the need for and role of routine genomic sequencing and molecular characterization in men with advanced PC.

Here, we describe the clinical case of a de novo mPCa, with the pathology and imaging results. In light of the current state-of-the-art, whole-exome sequencing and a transcriptome analysis of the tumor biopsy was performed. We discuss the genomic and transcriptomic findings in light of the known molecular mechanisms of PCa development and discuss the complexity of clinical decision-making for further disease medical management in a comprehensive approach incorporating both germline and somatic genomic testing.

## 2. Case History

**Figure 1:**
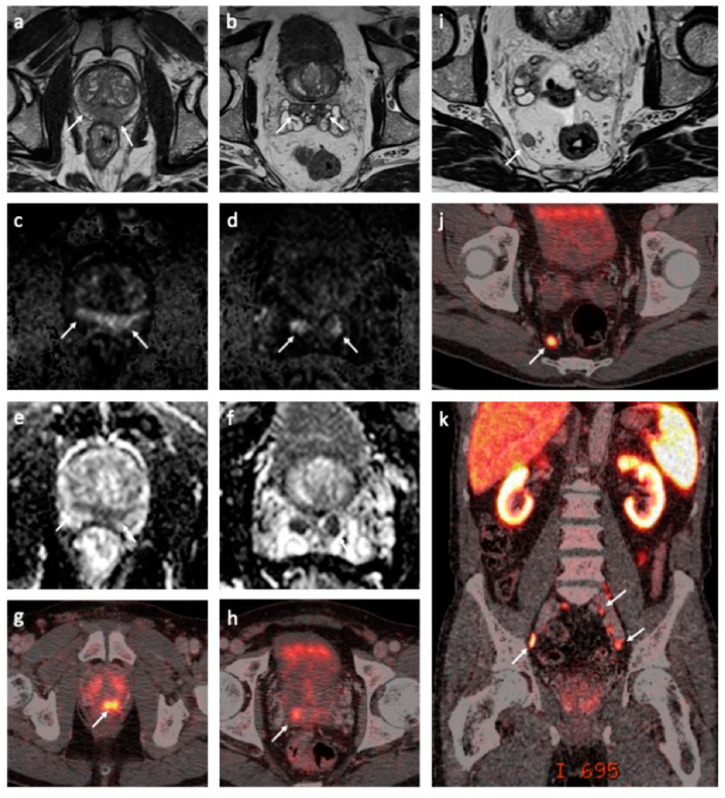
Magnetic resonance imaging (MRI) and prostate-specific membrane antigen (PSMA)-positron emission tomography (PET)/computed tomography (CT) findings at the baseline. Axial T2-weighted MRI sequences demonstrated a large, diffuse infiltrating tumor in the peripheral zone with seminal vesicle invasion ((**a**,**b**), white arrows). The tumor was hyperintense on diffusion-weighted MRI (DW-MRI) (b = 1500 s/mm^2^) ((**c**,**d**), white arrows) with a corresponding low apparent diffusion coefficient (ADC) (ADC = 0.6 × 10^−3^ mm^2^/s) ((**e**,**f**), white arrows). Corresponding tracer uptake on fused PSMA-PET/CT images ((**g**,**h**), white arrow). A large lymph node metastasis in the mesorectal fat is shown on the axial T2-weighted MRI ((**i**), white arrow) and fused PSMA-PET/CT images ((**j**), white arrow). A coronal-fused PSMA-PET/CT image demonstrates multiple metastases to the pelvic lymph nodes ((**k**), white arrows).

We report the case of a 68-year-old man who was diagnosed with de novo mPCa T3bN+M1a+b stage in November of 2019. The patient had no family history of PCa. The pathology report showed a Gleason score of 9, grade group 5 in all 10 biopsies at diagnosis. Morphologically, intraductal carcinoma was the dominant cell pattern, while cribriform and perineural growth were observed in the pathological examination [10]. The pretreatment prostate-specific antigen (PSA) level was 13 ng/mL. Multiparametric magnetic resonance imaging (mpMRI) and prostate-specific membrane antigen (PSMA)-positron emission tomography (PET)/computed tomography (CT) confirmed the tumor spread to the pelvic lymph nodes and a possible solitary metastasis to one of the thoracic vertebrae (Figure 1). The immunohistochemical analysis of primary tumor biopsies revealed the expression of all MMR proteins and was negative for PD-L1 staining but showed focal CD3 positivity.

The patient had low thrombocytes values. Further investigation of the cell morphology and flow cytometry of a bone marrow biopsy confirmed a diagnosis of hairy-cell leukemia. In addition, the same examination confirmed AE1/AE3 cytokeratin positivity and the pathognomonic marker NKX3.1 originally for epithelial-positive cells suspected to represent bone infiltrates of the prostate cancer.

**Figure 2:**
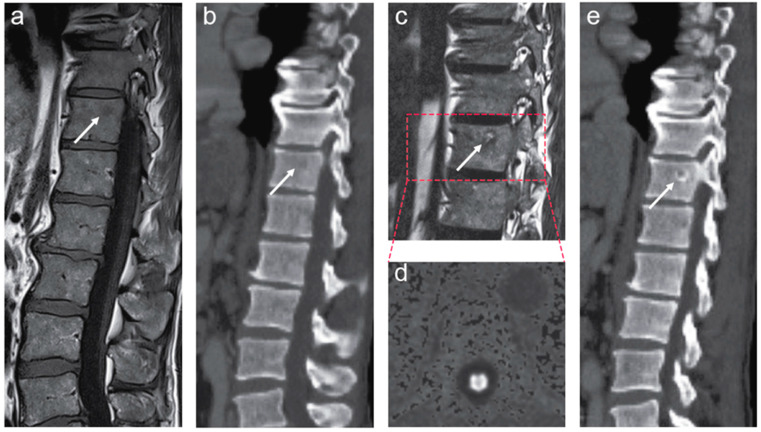
The left panel (**a**,**b**) shows pretreatment magnetic resonance imaging (MRI) and computed tomography (CT) scans of the thoracolumbar spine. Small, focal lesion in the thoracic spine is shown on MR ((**a**), white arrow) and CT ((**b**), white arrow) images. The right panel (**c**–**e**) shows follow-up MRI and CT performed after the start of combined androgen deprivation therapy. A sagittal T2-weighted fat-only Dixon image demonstrates an increased fat signal within the suspected lesion in the thoracic spine (**c**, white arrow). There was no evidence of tumor activity on the diffusion-weighted MRI (DW-MRI) (**d**). CT of the thoracolumbar spine showed subtle perilesional sclerosis ((**e**), white arrow). The findings (**c**–**e**) have appeared since the pretreatment MRI and CT scans and are presumably consistent with the treatment effect in solitary bone marrow metastasis.

Before the initiation of long-term ADT, new snap-frozen biopsies were sampled, stored in nitrogen, and sent for whole-exome and transcriptome sequencing. The obtained results were discussed within a tumor board. A combined treatment with ADT and radiotherapy was suggested. After four months of ADT, the total testosterone suppression was achieved by adding enzalutamide to goserelin during and after radiation. Due to COVID-19-related delays in the course of treatment, fiducial gold markers were installed in the prostate in late-April 2020, and image-guided conformal radiotherapy of 74 Gy to the prostate was applied from May to June 2020. The patient had a PSA nadir of 0.44 ng/mL at the end of the radiotherapy, and he was subsequently followed by his local oncologist. A follow-up MRI scan revealed signal changes on fat-only and water-only Dixon images and diffusion-weighted images (DWI) within the suspected lesion in the thoracic spine, presumably consistent with the treatment response of the bone marrow metastasis (Figure 2). Furthermore, a correspondingly good treatment response in baseline radiographically outlined lesions was revealed in the prostate tumor and lymph node metastases on follow-up MRI.

At the last follow-up examination, in August 2020, the PSA level fell to less than 0.03 ng/mL. ADT with goserelin and enzalutamide was continued. All procedures followed were in accordance with the ethical standards as required by national law and with the Helsinki Declaration of 1975 (in its most recently amended version). The patient consented to all the molecular studies and the publication of his case.

## 3. Materials and Methods

### 3.1. DNA and RNA Extraction

Original material: EDTA blood sample and needle biopsy material snap-frozen claimed to contain a significant tumor cell fraction. DNA was subsequently extracted, and measurements for the biopsies are listed in Supplementary Table S1 (blood DNA went through a precipitation prior to the measurements). The tumor biopsy and blood samples were subject to whole-exome sequencing using the Twist Human Core Exome Plus kit (Twist Bioscience, San Francisco, CA, USA) on a NovaSeq 6000 sequencing instrument (Illumina, San Diego, CA, USA). Read length was 2 × 100 basepairs. The needle biopsies were also used for library preparation of RNA sequencing.

### 3.2. Whole-Exome Sequencing Analysis

A bioinformatics pipeline was applied to identify the acquired single-nucleotide variants and short insertions and deletions (indels) in the tumor sample. DNA sequence reads from the blood (control) and tumor samples were initially aligned to the human reference genome (build b37 with an added decoy contig) using BWA-mem v0.7.15 [11]. Read duplicates were marked with Picard tools (v.2.5.0), and GATK tools (v3.7) were further applied for a two-step local realignment around indels, recalibration of the base quality, and calculation of the coverage statistics [12]. Somatic SNV detection was performed with MuTect and Strelka, while Strelka was used for the identification of indels [13,14]. Variants with a sequencing depth in the tumor below 10 were considered unreliable and not used for the downstream analysis.

FACETS (version 0.5.0) was used for copy number identification, in which allele-specific copy numbers are corrected for tumor purity, ploidy, and clonal heterogeneity [15]. Genomic regions with loss or gain were identified using a log2 threshold of +/− 0.8. 

For variant interpretation and functional annotation, we utilized the Personal Cancer Genome Reporter (PCGR v0.9.0) [16]. For the assessment of microsatellite instability (MSI) status (MSI-H vs. MSS), we used the statistical classifier integrated with PCGR, which was trained with somatic variant data from TCGA tumor samples. The estimated contribution of mutational signatures (single-base substitutions, SBS) was performed with MutationalPatterns (v2.0.0) using the reference collection of *n* = 67 signatures (COSMIC v3) [17,18]. We restricted the signature fitting to the reference signatures previously attributed to PCa (SBS1, SBS3, SBS5, SBS18, SBS37, SBS40, SBS41, and SBS58), as recommended [17].

Germline variants (SNVs/InDels) were identified with Illumina’s DRAGEN pipeline (software version 01.011.308.3.3.11) on the existing read alignment of the control (blood) sample. We investigated the germline variant set in the context of an exploratory, virtual panel of *n* = 216 protein-coding genes of relevance to cancer predisposition (Supplementary Table S2), using the Cancer Predisposition Sequencing Reporter v0.6.0 [19].

### 3.3. RNA Sequencing and Analysis

Libraries were prepared using the TruSeq Stranded mRNA kit (Illumina, San Diego, CA, USA) according to the manufacturer’s instructions and sequenced with the NovaSeq 6000 System (Illumina, San Diego, CA, USA). All analyses were performed through shell scripts and R (https://www.r-project.org/ (accessed on 1 September 2020)) using Bioconductor (https://www.bioconductor.org/ (accessed on 1 September 2020)) packages. Raw RNA sequence reads (in the fastq format) were checked for the quality of sequencing through FASTQC (https://github.com/s-andrews/FastQC (accessed on 1 September 2020)). Trimmomatic (v0.38) was used for read trimming [20]. Reads were mapped to the human reference genome (GRCh38) using STAR v2.7.0 with the default parameters [21]. GENCODE was used for transcript annotation (version 22; the same version that was used for RNA-seq data processed within TCGA). Reads per transcript were estimated with the option *quantMode GeneCounts* in STAR. Transcript abundance levels were finally transformed into normalized expression units in the form of TPM (transcripts per million). Activity levels for gene expression signatures were evaluated through a gene set variation analysis (R package *gsva*) using log-transformed TPM values pr. gene as the input.

We used RNA-seq data from The Cancer Genome Atlas (TCGA, release 28) as a reference distribution for the gene expression levels in tumor samples. Specifically, we downloaded gene expression data for the primary tumor samples using the TCGAbiolinks package [22] and converted the expression unit from FPKM to TPM values. 

For the detection of fusion transcripts, Arriba (v2.1.0) and STAR-fusion (v1.9.1) were used with the default parameter settings [23,24]. GRCh38 was used as the reference genome and GENCODE (v22) as the gene transcript model during read mapping and fusion calling. In the output of Arriba, the quality of fusion transcript callings was evaluated using a confidence tag (e.g., high, medium, and low), which suggests the likelihood that a fusion candidate is robust rather than an artifact. To improve the sensitivity of detection, the nominated fusion candidates by Arriba and STAR-fusion were merged.

### 3.4. Immunohistochemistry

The immunohistochemistry of the sections from formalin-fixed paraffin-embedded prostate carcinoma tissue was performed by mouse anti-MSH2 (clone G219-1129, Ventana, Oro Valley, AZ, USA,; “ready to use”), rabbit anti-MSH6 (clone EP49, Epitomics, Burlingame, CA, USA, dilution 1:50), and mouse anti-MLH1 (clone G-168-15, BioCare, Pacheco, CA, USA, dilution 1:50) for MMR proteins. For PD-L1, mouse anti-PD-L1 (clone 22C3, “ready to use”) was used. In all specimens, the presence of MMR proteins and lack of expression of PD-L1 was confirmed.

## 4. Results

### 4.1. Genomic Findings—Tumor

**Figure 3:**
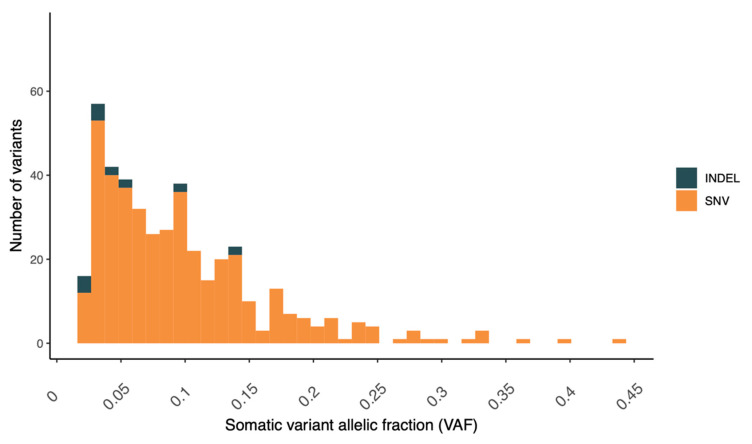
Distribution of the variant allelic fraction for somatic variants (SNVs/InDels) detected in the prostate tumor case.

The tumor cellularity or purity of the biopsy, as estimated from FACETS, was found to be relatively low (30%). Low purity will reduce the effective coverage of variant alleles in the tumor cells, in turn reducing the detection sensitivity [25]. As a consequence of the low tumor purity, the distribution of variant allele fractions (VAFs) was skewed towards the lower end (Figure 3).

A total of 149 protein-coding variants were detected, and the tumor mutational burden (TMB) was calculated accordingly as 3.88 (nonsynonymous variants pr. Mb), which may be somewhat uncertain, given the low tumor content in our case (the complete set of variants are listed in Supplementary Table S3), yet a high TMB for primary PCa. It has been estimated that the TMB of unselected and usually treatment-naïve locoregional prostate adenocarcinoma cohorts typically falls between 0.94 and 1.74 nonsynonymous mutations per megabase [26]. 

The copy number analysis performed with FACETS did not reveal any regions with significant amplifications or homozygous deletions.

**Figure 4:**
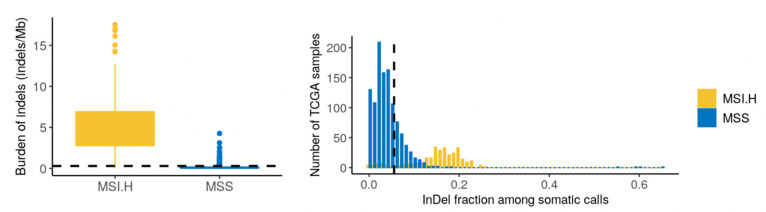
Assessment for the potential microsatellite instability (MSI) of the tumor case through a somatic variant comparison with TCGA samples with a verified MSI status (as detected with a PCR-based assay of mono- and dinucleotide repeats). The value for the prostate tumor case is depicted by a black, dashed line. MSS = microsatellite stable tumors (TCGA) and MSI.H = microsatellite instable tumors (TCGA).

MSI is a result of impaired DNA mismatch repair and constitutes a cellular phenotype of clinical significance in many cancer types [27]. We utilized the patterns and load of indels in the tumor to examine the MSI status (MSI-high vs. MSS). Specifically, we applied a machine-learning classifier trained on tumor samples in TCGA (breast, ovarian, stomach, and endometrial) with established MSI status from mononucleotide repeat assays. The tumor was classified as microsatellite stable (MSS), with properties resembling microsatellite stable tumors (Figure 4). Furthermore, no somatic alterations were detected in the mismatch repair genes.

We estimated the contribution of mutational signatures by computationally fitting established signatures (COSMIC v3) to the patterns of nucleotide variants found in the tumor biopsy. The accuracy by which the mutational profile could be reconstructed with reference signatures previously attributed to PCa (i.e., the fitting procedure) was 79%, indicating that the reference signatures cannot explain the mutations observed in an optimal manner. Mutational signature SBS3 was by far the most dominant signature, with a relative contribution of 59% (Supplementary Table S4). This signature is a relatively flat and featureless base substitution signature associated with defective homologous recombination-based DNA repair and inactivating mutations of *BRCA1* and *BRCA2* [28,29]. It is usually accompanied by small deletions, with overlapping microhomology at their boundaries and large numbers of rearrangements, including tandem duplications and deletions [28].

### 4.2. Somatic Variants of Potential Clinical Relevance

#### 4.2.1. PTEN Inframe Deletion—p.His196_Ile203del

We revealed a 24-base pair interstitial deletion causing an in-frame deletion of seven amino acids from the *PTEN* protein. The deletion was found with a very low allelic fraction (2.8%), indicating that it may represent a subclonal event. Inactivation of the *PTEN* tumor-suppressor gene by deletion occurs in 20–30% of PCa tumors, and a loss of *PTEN* function is strongly associated with a poor outcome [30]. In a study of TCGA prostate samples, *PTEN* loss-of-function not only led to activation of the PI3K/AKT pathway but, also, affected the genome stability and levels of tumor aneuploidy, especially in cases with *PTEN* homologous deletion. PTEN-deficient cells exhibit elevated levels of reactive oxygen species, increased endogenous DNA damage, and constitutive *ATM* activation. *ATM* inhibition (discussed below) has been reported to be specifically toxic to PTEN-mutant cancer cells, thus providing a mechanistic rationale for a clinical evaluation of the inhibitors in PTEN-deficient tumors [31]. The pHis196_Ile203 inframe deletion we discovered is not, however, a classic loss-of-function variant caused, e.g., by frameshift and was not found in the COSMIC database of somatic mutations in cancer or in tumor samples in the TCGA database.

#### 4.2.2. ATM Missense Variant—p.Arg1575His 

A somatic missense mutation in *ATM* was identified with an allelic fraction of 13.6%. Germline *ATM* mutations have been found to increase the risk of developing PCa (among other cancers) [32]. Interestingly, this particular variant has also been observed as a germline variant and is listed as a variant of unknown significance (VUS) in ClinVar (accession identifier RCV000159727.9). The *ATM* gene encodes a PI3K-related serine/threonine protein kinase that plays a central role in the response to, and ultimately the repair of, DNA double-strand breaks (DSBs). Once activated, *ATM* phosphorylates multiple substrates, protein kinases, and sensor proteins in order to carry out DSB repair and, also, regulate the normal cell cycle processes, such as apoptosis and checkpoint activation. Further, *ATM* germline mutations have been linked to an increased level of sensitivity to platinum-based antineoplastic drugs [33].

#### 4.2.3. CREBBP Missense Variant—p.Trp1718Gly 

The *CREBBP* variant we identified is a missense variant also with a very low allelic fraction (3.5%), possibly indicating its presence in a clone. Targeting the CBP/p300 bromodomain has shown therapeutic potential in castration-resistant PCa [34]. A recent report showed how a small molecule inhibitor (CCS1477) can decrease the influence of the coactivator p300/CBP on AR activity in castration-resistant PCa [35].

### 4.3. Germline Findings—Blood

Using an exploratory virtual panel of *n* = 216 genes of relevance for cancer predisposition, we examined the case for pathogenic germline variants (details for the panel are listed in Supplementary Table S3). We identified a total of *n* = 111 protein-coding variants in these genes, but none of them were classified previously as pathogenic or likely pathogenic (P/LP), according to ClinVar, and none of the novel (i.e., not recorded in ClinVar) variants were classified as P/LP by the algorithm used in CPSR. A total of *n* = 14 variants were classified as variants of uncertain clinical significance. A few of these variants were in genes involved in DNA repair and are described briefly below (details per variant are provided in Supplementary Table S5).

A missense variant in *BARD1* (BRCA1-associated RING domain protein 1) was detected, a variant that has been reported previously for hereditary cancer conditions in ClinVar (NM_000465.4:p.His116Tyr, rs144856889). *BARD1* is a cancer-susceptibility gene that interacts with *BRCA1* in homology-directed repair (HDR). A recent study investigated the functional impact of numerous *BARD1* missense variants on HDR proficiency and discovered that HDR-deficient variants were located in distinct functional domains [36]. Although the His116Tyr variant found in our case was not assessed in that study, it was located in close proximity to a variant that was reported to have no impact on the repair proficiency (p.Asn118Ser). Thus, it seems likely that the His116Tyr variant does not affect the proficiency of homologous recombination repair (HR). 

A germline splice region variant was found in *MSH2*, previously reported for Lynch syndrome conditions in ClinVar (rs779102258). However, this variant was found to be located in a poly-A tract and may well be an artefact from sequencing or read alignment. We also discovered a missense variant in *ERCC5* (NM_000123.4:p.Gly1080A, rs9514067); however, this gene is not directly involved with homologous recombination repair but, rather, nucleotide excision repair.

### 4.4. Transcriptomic Findings—Tumor

The transcriptome of the tumor case was characterized with high-throughput RNA sequencing. Initially, we verified that the gene expression patterns from our tumor biopsy resembled the signals previously shown for prostate tumor samples. Specifically, using the expression profile for a set of *n* = 89 protein-coding genes linked to PCa development (gene set KEGG_PROSTATE_CANCER in MSigDB [37]), a Spearman rank correlation was calculated between our case and all primary tumor samples (*n* = 9346) in TCGA. Samples originating from the prostate adenocarcinoma cohort (TCGA-PRAD) were heavily enriched among the samples showing the strongest correlation to our case (96.4% prostate samples among the 500 cases with the strongest correlation).

**Figure 5:**
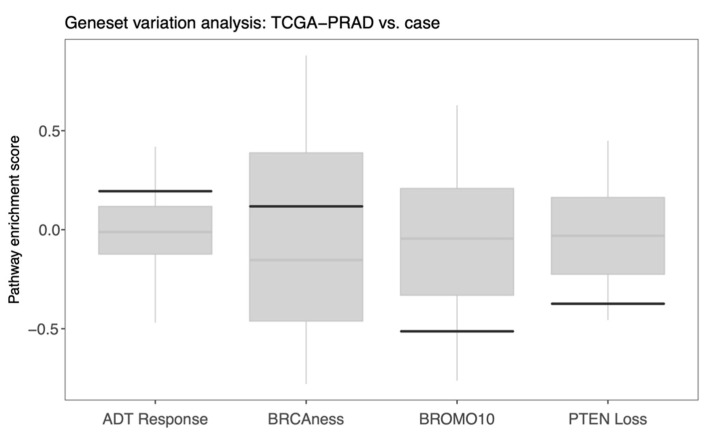
Gene set variation analysis of gene signatures attributed to ADT response, HR deficiency/BRCAness, response to bromodomain inhibitors (BROMO10) and aberrant PTEN tumor-suppressor pathway activity. Signature enrichment scores were calculated for all the prostate adenocarcinoma samples in TCGA (Gleason score >= 8, *n* = 201). In the boxplots, scores for our case are indicated with solid black lines, and median scores in TCGA-PRAD are indicated with gray horizontal lines.

The use of RNA-based molecular classifiers to stratify PCa patients for the risk of relapse to primary or secondary treatment is becoming an attractive clinical strategy [38]. Therefore, we interrogated a few published gene expression signatures related to disease progression and molecularly targeted treatment regimens for our case (Supplementary Table S6). In particular, we investigated a BRCAness signature (HR deficiency, *n* = 10 genes), a signature with 49 genes that correlate with response to ADT, a signature associated with response to bromodomain inhibitors (*n* = 10), as well as a signature of aberrant *PTEN* tumor-suppressor pathway activity (*n* = 190 genes) [39,40,41,42]. As our case presented a Gleason score 9, we used expression data from prostate adenocarcinoma samples in TCGA (Gleason score >= 8, *n* = 201 samples) and performed a gene set variation analysis in order to evaluate whether the PCa case had relatively high or low scores for these signatures. A strong positive enrichment score was observed for the signature associated with ADT response (Figure 5). The signature attributed to *PTEN* pathway activity showed a considerable negative enrichment score in our case, indicating a downregulation of this pathway. The bromodomain signature, a proxy for response to BET inhibitors, also showed a significant negative score. The evidence for BRCAness appeared with a modest positive signature enrichment score and above the median for the primary prostate tumor samples in TCGA.

### 4.5. Fusion Transcript Findings—Tumor

**Figure 6:**
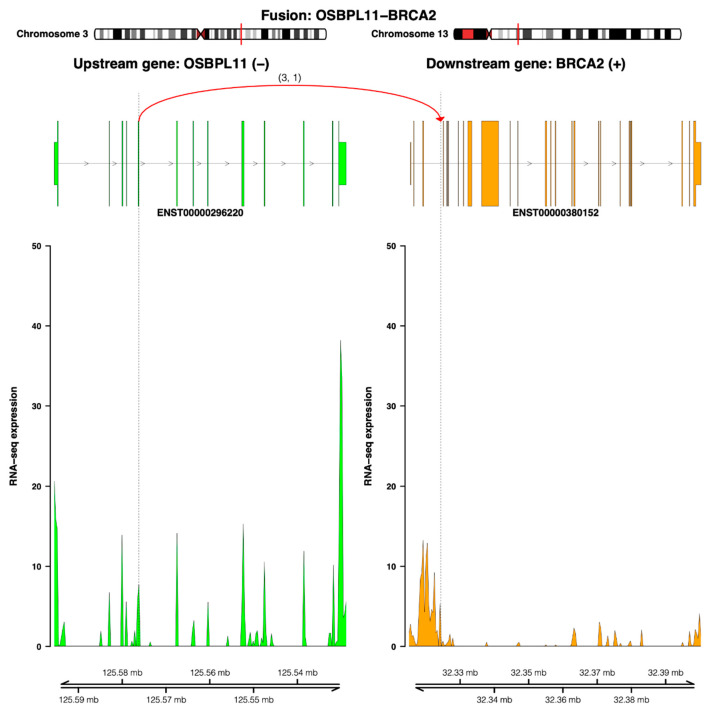
The *OSBPL11-BRCA1* fusion transcript. The genomic view of the fusion event is from the top showing breakpoint positions in a chromosome ideogram, the fusion linking exon 5 of the upstream partner *OSBPL11* (*ENST00000296220*—colored by green) to the intron (between exons 3 and 4) of the downstream partner *BRAC2* (*ENST00000380152*—colored by orange) and the number of split and discordant reads supporting the fusion (curved line), the RNA expression levels (read coverage), and genomic coordinates of the partner gene loci in Mb from the chromosome.

The fusion transcript analysis of RNA-seq data resulted in a list of 18 candidates, of which seven were tagged as high-confident candidates (Supplementary Table S7). Two novel high-confident fusion transcripts involved partner genes with considerable molecular relevance in PCa. The first one, an *OSBPL11-BRCA2* fusion transcript, was predicted to have an out-of-frame open reading frame (ORF) without encoding a chimeric protein. The breakpoint was located at the intronic region of *BRCA2* and did not match the intact conserved exon–exon boundaries. Figure 6 illustrates how the read coverage of downstream exons at the *BRCA2* breakpoint site are reduced compared to the upstream exons, indicating reduced expression.

The second relevant fusion transcript involved *PIK3R1*, which encodes a key regulatory subunit of the phosphoinositide-3 kinase (PI3K) pathway. The *PIK3R1-HSD17B4* fusion was the only nominated fusion transcript that could form an inframe ORF encoding a chimeric protein (Supplementary Figure S2). The putative fusion protein combines the SH3 and RhoGAP domains of *PIK3R1* with MaoC-like and SCP2_sterol-bd domains of *HSD17B4*.

## 5. Discussion

For mPCa, the Philadelphia and NCCN guidelines recommend germline and somatic testing within a priority gene panel covering *MLH1, MSH2, MSH6*, and *PMS2* (for Lynch syndrome) and homologous recombinant genes *BRCA1/2, ATM, PALB2*, and *CHEK2.* This recommendation is based on mutation prevalence and existing treatment options. The challenge ahead will be to define the standardized methods to decipher the information accumulated for each case and convert it to clinical treatment strategies. This can only be achieved by a concerted approach involving multidisciplinary teams to bring home knowledge for the benefit of our patients [43]. For the case presented here, interdisciplinary discussions were undertaken to evaluate the possible treatment options.

### 5.1. Pathology Findings

In a comprehensive approach, the pathology report (mainly intraductal/cribriform and grade 5 cancer) suggests an aggressive tumor [10]. No neuroendocrine differentiation was reported. The evidence suggests that intraductal PCa are heterogeneous, and some of these respond to ADT [44]. Tumor cell deposits had been found in the bone marrow of the patient advocating for both maximal androgen deprivation and early stereotactic radiotherapy [45]. Concordantly, targeting the androgen receptor (AR) axis with enzalutamide and goserelin led to an observed PSA decline below 0.03 ng/mL. The remarkable response to maximal androgen deprivation could indicate a preferential androgen-driven pathway, as elucidated by Zhao et al. [46]. Using a PAM50-based gene expression subtype classifier, patients were neatly dissected into three different major response types, and one of those, the luminal B type, was clearly related to the clinical benefit of ADT.

### 5.2. MRI Findings

MRI is considered the most sensitive and specific imaging technique for localizing and staging clinically significant PCa. In our patient, the pretreatment imaging findings, including low apparent diffusion coefficient (ADC) values within the tumor, were consistent with the features of aggressive PCa and are in agreement with previous reports [47].

### 5.3. Genomic and Transcriptomic Findings

The molecular findings at the genomic and transcriptomic levels were interrogated specifically with respect to the treatment options. Some novel biomarkers for prognosis and diagnosis, beyond the Gleason score and PSA level, have been proposed for prostate cancer management, but these were not investigated in detail for our case [48,49].

The incidence of pathogenic germline mutations in DNA repair genes in men with mPCa has been estimated to be almost 12% [50]. Having considered a large set of known cancer predisposition genes, we could not identify any pathogenic germline mutation in our case. Nonetheless, two potentially actionable targets could be identified from the tumor sequencing analysis of the patient. Somatically acquired protein-coding alterations in *PTEN* and *ATM* were identified in the tumor biopsy. The *PTEN* somatic alteration was accompanied by a concomitant suggested loss of *PTEN* activity by the transcriptome analysis. Interestingly, the somatic *ATM* variant (p.Arg1575His) has been reported multiple times as a germline variant and is currently classified as a VUS in ClinVar. Alterations in MMR genes could not be confirmed at the protein level, and the transcriptome analysis showed a relatively low BRCAness score. However, the large contribution of the mutational signature SBS3, signaling a HR defect, indicated that the *ATM* variant may be implicated in the mutational processes acting on the tumor. Furthermore, the presence of an *OSBPL11-BRCA2* fusion transcript points to a functional loss of *BRCA2*, a gene that regulates the activity of *RAD51*, an essential protein for proficient HR [51]. The differential expression of exons before/after the breakpoint in the *BRCA2* gene indicated that one copy of *BRCA2* was broken up by this fusion event.

Some limitations of our genomic approach must be acknowledged. First, the yield of DNA extracted from the tumor was low, possibly introducing a possible bias in our assessment of low TMB. Second, the MSI classifier applied in our analysis has not yet been validated by samples from PCa patients. Additionally, we observed that the reference mutational signatures that were fitted to the mutations in the tumor could not optimally explain the patterns observed. Notably, the presence and contribution of “flat” signatures, such as the mutational signature SBS3, attributed to HR deficiency, are generally hard to fit and estimate robustly [52].

### 5.4. Specific Treatment Considerations

Based on the strong pathology evidence for combining radiation with ADT, the patient has been treated with goserelin, and conformal radiotherapy was applied to the prostate [3,4]. Since AR was reported to maintain the expression of DNA repair genes, and given the *ATM* mutation in our case, we added treatment with Enzalutamide following an enforced ADT during radiation to personalize the treatment [53,54]. Enzalutamide prolongs survival when added to ADT and has shown clinical benefits as a first-line treatment in men with both nonmetastatic and castration-resistant metastatic disease [55,56]. The final results of the ENZAMET trial showed an overall survival at 3 years of 80% in the enzalutamide group, as compared to 72% in the standard-care group. With level 1 evidence supporting the superiority of enforced ADT, it was reasonable to involve this strategy, especially in the case of putative HR deficiency.

#### 5.4.1. PARP Inhibitors

PARP inhibitors (PARPi) are approved for biomarker-selected or unselected male patients with metastatic castration-resistant PCa with DNA repair deficiencies. The PROfound trial investigated the impact of olaparib in biomarker-selected (*BRCA1*, *BRCA2*, or *ATM*) patients [57]. The trial showed a progression-free survival benefit of 3.1 months in the olaparib arm (median PFS 7.4 months in the olaparib group compared to 3.6 months in the control group). The drug prolonged patient survival to a clinically significant median of 18.5 months compared to 15.1 months in the control group. Our patient had a somatic alteration in the *ATM* gene. In the context of cancers with deficiencies in homologous recombination (HR) repair, the association with indels, especially with microhomology at the breakpoint, makes mechanistic sense, since this presumably occurs by error-prone nonhomologous end joining (NHEJ) and the alternate-EJ. The *ATM* gene promotes NHEJ for DNA double-stranded break repair, but when deficient, the aberration-prone single-stranded DNA repair substitutes NHEJ. Thus, it seems plausible that *ATM* may be a reason for the observed mutational signature 3 in the tumor sample of our case and possibly may play a role in the relatively high TMB (compared to other prostate cancers).

In our case, the somatic *ATM* mutation, combined with the presence of mutational signature 3 (homologous recombination deficiency), could indicate a biomarker-based selection of our patient for strategy utilizing PARPi as monotherapy [58]. Moreover, ADT can induce a DNA damage repair-deficient phenotype, thereby promoting the effect of PARPi. Several ongoing studies (TALAPRO2 and MAGNITUDE) are investigating the role of PARPi in combination with a novel ADT in biomarker-unselected patients.

The BRCA2 fusion variant detected is particularly interesting in the context of PARPi treatment, in that phase 2 trials have shown that antitumor activity with PARPi in patients with metastatic mPCa have consistently higher response rates among those with BRCA2 alterations than those for other DNA repair gene alterations [57,59,60,61].

Our patient had hairy cell leukemia with an initially reduced platelet count. Personalized medicine drives the decision focus from assessing symptom-caused medicine to anticipating the disease development. Since PARPi administration is associated with thrombocytopenia and anemia, the suitability of cladribine administration to the patient was discussed with a hematologist. However, cladribine was not recommended due to the often-indolent course of hairy cell leukemia. So far, no agreement has been reached on the optimal clinical approach in a patient with two different diseases. Still, olaparib, a PARPi, is regarded as a possible first-line drug for the patient and was discussed interdisciplinarily.

We decided to challenge the hematologist’s view and, for the time being, postponed the PARPi intervention, supported by the favorable treatment response and absence of further tumor growth or detectable activity on follow-up MRI. In the case of oligo-progression, stereotactic radiotherapy to the thoracic bone lesion could be an alternative treatment to stretch the need for further systemic treatment [47].

On the other hand, combined PARPi/ATR access is under early clinical testing and might open up a novel avenue of treatment that circumvents adapted *ATM* resistance. Lloyd et al. recently showed that a combination of olaparib and an *ATR* inhibitor potentiated each other and led to accelerated cell death in a mouse model [58]. Future studies will show if this approach could be beneficial in mPCa. Following the Philadelphia guidelines, priority genes to test for metastatic disease treatment include germline variants in *BRCA1, BRCA2*, and mismatch repair genes, along with broader testing, such as *ATM*, for clinical trial eligibility. Recently, a first-in-humans trial with the *ATR* inhibitor BAY1895346 was conducted in 21 patients with advanced solid tumors [62]. If this successfully progresses to phase 2 trials, this would also be a possible option for our case.

#### 5.4.2. Targeting Consequences of Loss of PTEN Activity

Loss of the tumor-suppressor *PTEN* function, a major finding in men with mPCa is associated with poor outcomes [63]. The transcriptome analysis showed a low activity of the *PTEN* tumor-suppressor pathway. However, the PTEN deletion appears to be present only at a subclonal level in our case. We have therefore developed an assay for this particular *PTEN* deletion as a circulating-tumor DNA marker for further detailed monitoring of the treatment response and detection of treatment resistance based on the potential expansion of this clone (see section *PTEN deletion monitoring assay* in Supplementary Materials). To this stage, we have not detected clonal expansion of the clone harboring the PTEN deletion.

#### 5.4.3. HDAC Inhibitors

HDACi have been shown to synergize with PARPi and DNA-damaging agents through multiple mechanisms, including the induction of DNA damage, attenuation of HR protein *ATM* activity, inhibition of both NHEJ and HR systems, induction of p53 acetylation and activity, downregulation of antiapoptotic proteins, and upregulation of proapoptotic proteins [64].

#### 5.4.4. Immune Checkpoint Inhibitors

According to the preliminary results of an ongoing trial, PCa patients with tumors having a PD-L1 level of at least 1%, HR deficiency mutations, DNA damage repair mutations, or a TMB greater than the median of 74.5 mutations showed enhanced responses to a combined nivolumab/ipilimumab treatment [65]. Our subject has signs of HR deficiency (the most dominant mutational signature), an ATM mutation of unknown clinical significance, a *BRCA2* fusion transcript that likely reduces full BRCA2 functionality, and a significantly higher TMB than the median for PCa [26]. BRCA2-altered PCa tumors have been shown to harbor enhanced intra-tumoral immune infiltrates compared to wild-type tumors [60], suggesting treatment options targeting immune cell modulation [66]. The relatively high mutational load and CD3 influx could indicate a possible response if the patient is challenged with checkpoint inhibition (CPI). Nevertheless, to date, the attempts to treat mCRPCa with pembrolizumab, a checkpoint inhibitor, have not been very successful [67]. Furthermore, although pembrolizumab CPI-agnostic therapy has been approved for MSI-high tumors, our case presented an expression of all MMR proteins (IHC), and PD-L1 staining was negative. Of note, mismatch repair deficiency has been shown to be a very uncommon phenomenon in PCa, with a reported prevalence of only 3% [68]. Subudhi et al. showed that patients with high TMB, high T-cell density, and a high IFN-gamma response signature had favorable outcomes on the CPI [69]. The primary biopsies in our case showed only the focal presence of CD3. ADT can reverse thymic involution, thereby recruiting naïve T cells capable of forming lymphocyte infiltrates in the primary tumor [70,71]. Of note, the typical immune-pathological cell picture is governed by a suppressed immunity for PCa patients when treated with ADT [72]. In summary, this evidence discourages instantly a first-line treatment with CPI monotherapy. However, findings from a phase 1 trial of a therapeutic peptide vaccine in men with de novo mPCa could overcome the immunosuppressive tumor microenvironment, indicating an interesting combined approach that will be further evaluated [73] or BRCA2-induced immune cell modulation [66].

#### 5.4.5. Current Preferred Considerations for the Management of the Patient

The heterogeneous findings from genomic testing can potentially evoke further actions for our case:

- Intraductal carcinoma with grade group 5 should encourage participation in clinical trials.
- Continuous PTEN deletion as the biomarker-based monitoring of treatment efficacy in liquid biopsies, which could define a switch into therapies targeting the mTOR/PI3K/AKT axis.
- Treatment with PARPi in combination with an AR blockade.
- Immune modulation.
- Test of the ATM variant in preclinical models for the response to a treatment with PARPi and AKTi.
- Stereotactic radiotherapy to a single thoracic lesion.

## 6. Conclusions

The clinical case illustrated here pinpoints the many challenges inherent in the decision-making process for personalized treatments. Although the genomic findings provided a comprehensive understanding of the complex scenario ahead in managing this patient, we adopted a treatment strategy in compliance with the existing guidelines.

## Data Availability

All processed molecular data of the tumor biopsy and R code for the transcriptome analysis are available through the following DOI: https://doi.org/10.5281/zenodo.4596571.

## Supplementary Materials

The following are available online at https://www.mdpi.com/2075-4426/11/5/330/s1: Figure S1: Amplification products from two PCR assays of the PTEN deletion, Figure S2: The *PIK3R1-HSD17B4* fusion transcript, Table S1: DNA extraction measurements, Table S2: Germline screening panel, Table S3: Somatic variants, Table S4: Mutational signatures, Table S5: Selected germline variants of uncertain clinical significance, Table S6: Gene expression signatures, Table S7: Fusion transcripts, Table S8: PTEN deletion monitoring assay primers.
