# Supplementary material for: The Quandary of DNA-Based Treatment Assessment in De Novo Metastatic Prostate Cancer in the Era of Precision Oncology
Source: J Pers Med. 2021 Apr 22;11(5):330. doi: 10.3390/jpm11050330 (PMC8143497; doi:10.3390/jpm11050330)
Supplement: Supplementary file 1 [file jpm-11-00330-s001.zip › jpm-1161439-supplementary/PCa_Case_Report_Proof_20210422_Supp.pdf]

Supplementary Materials

PTEN deletion monitoring assay

The 24 base pair deletion in PTEN detected by sequencing (*chr10:g.89711965TTTCACAAGATGATGTTTGAAACTA>T* (grch37)) was verified with an independent mismatch amplification assay. By use of a common forward primer and two reverse primers, wild type DNA and DNA with the deletion was amplified (Supplementary Table 8). The amplified PCR products were separated by length in a capillary electrophoresis instrument. The common forward primer was tail with an 18 base pair sequence which allowed for attachment of desired fluorescent molecules. The total lengths of the amplicons were 208bp and 189bp for wild type and mutant, respectively. The assay was used to amplify DNA from primary tumor, white blood cells, and cell-free DNA from plasma. Six technical replicates were amplified for each primer and template sets. Representative peak detection is shown in Supplementary Figure 1.

The PCR reaction mixture contained 1 µl of extracted DNA (from tumor or WBC) or 3µl cell free DNA, 0.4 mM dNTPs (0.2 mM of each dNTP) (VWR, Oslo, Norway), 1× Thermopol Buffer, 2 mM MgSO4, 0.075unit Taq/µl, 0.15 µM of each forward, reverse and fluorescently labelled primer (Integrated DNA Technologies, Leuven, Belgium) and total reaction volume of 10 µl. The temperature cycling was performed in a Eppendorf Mastercycler ep gradient S (Eppendorf, Hamburg, Germany) with an initial denaturation 94 °C for 60 sec followed by cycling 35 or 38 times under the following conditions, denaturation at 94 °C for 15 sec, annealing 57 °C 20 sec and elongation at 72 °C for 40 sec.

Full blood collected in EDTA vacutainer (Becton Dickinson) was quickly centrifuged to separate blood cells from the plasma. The plasma layer was transferred to new tubes and subjected to a second centrifugation. Eight milliliter of the top plasma layer from the second

centrifugation was used for cell free DNA extraction. The DNA was extracted using QIAamp DSP Circulating NA Kit (Qiagen) according to the manufacturer's recommendation.

Amplification products were visualized by capillary electrophoresis in MegaBACE 1000 DNA Analysis System (GE Healthcare Life Sciences, Pittsburgh, PA). Internal standard and samples were separately injected into the capillaries from 96-well plates by electrokinetic injection at 161 V/cm for 60 sec. The temperature of the capillary chamber was set to 27 °C and electrophoresis was carried out at a constant field of 145 V/cm. The sieving matrix used was linear polyacrylamide (Gel Company, San Francisco, CA). The cathode and anode side of the capillaries were submerged in buffer (30 mM tris, 0.1M TAPS, 1.0 mM EDTA, pH 8.00) during electrophoresis.

Supplementary Figures

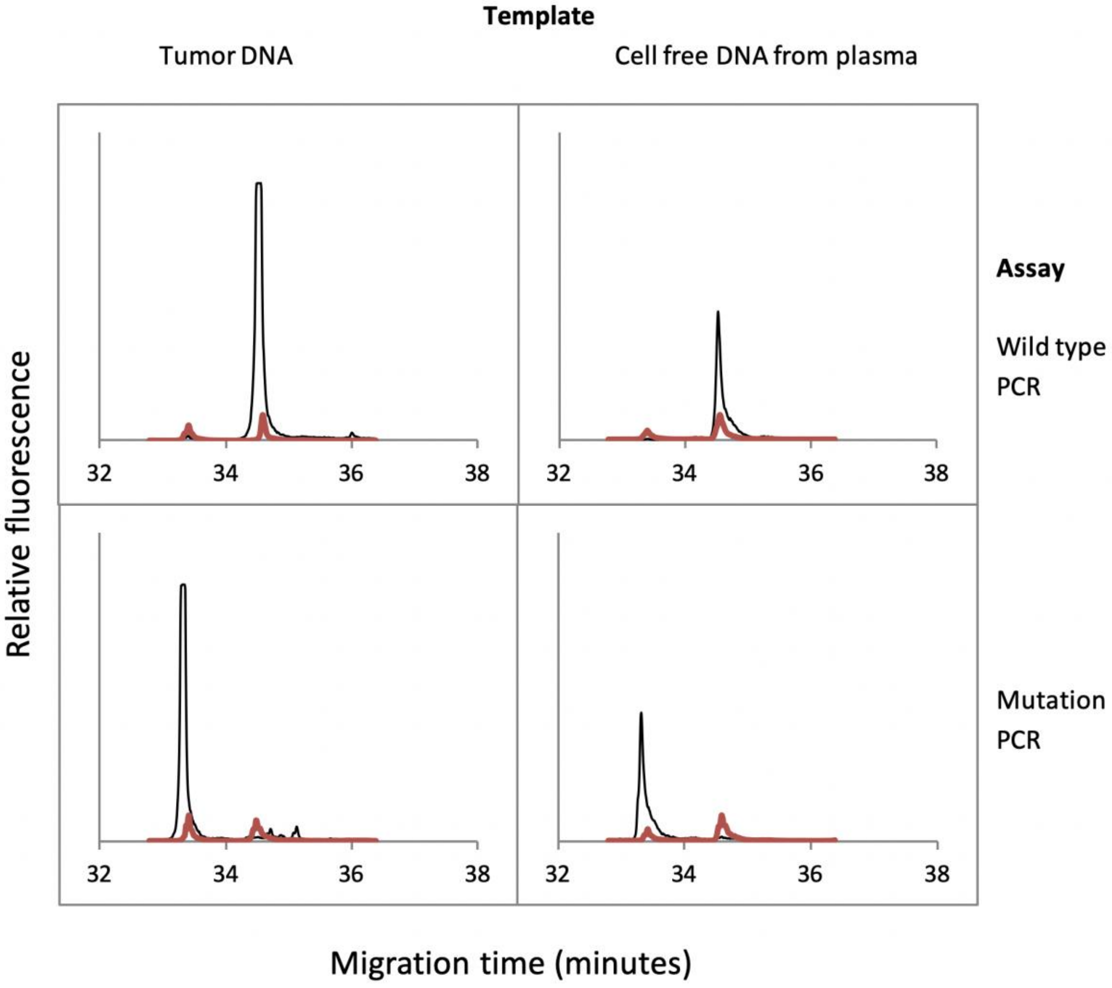

Supplementary Figure S1: Amplification products from two different templates and the two PCR assays of the PTEN deletion. Black peaks; sample signal. Red peaks; internal standard (532 ATTO labeled) with fragment size of 189 (first peak) and 208 (second peak) base pairs, respectively. Electropherograms are aligned with respect to the internal standard.

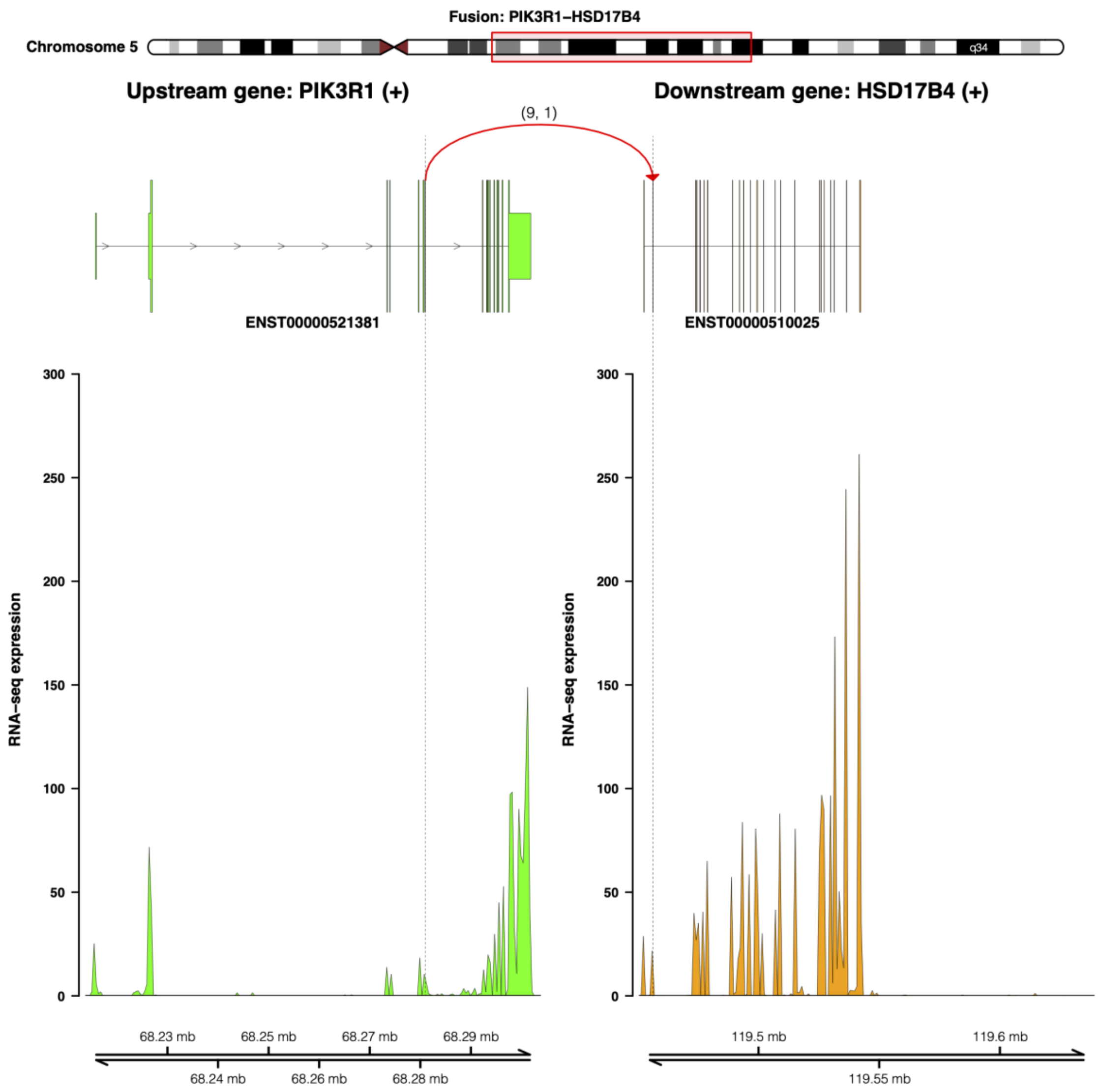

Supplementary Figure S2: The *PIK3R1-HSD17B4* fusion transcript. The genomic view of the fusion event is from the top showing breakpoint positions in a chromosome ideogram, the fusion linking exon 7 of the upstream partner *PIK3R1* (*ENST00000521381* – coloured by green) to the exon 2 of downstream partner *HSD17B4* (*ENST00000510025* – coloured by orange) and the number of split and discordant reads supporting to the fusion (curved line), the RNA expression levels (read coverage), and genomic coordinates of partner gene loci in Mbp from the chromosome.

Supplementary Tables

Table S1 - DNA sample extraction measures

Table S2 - Somatic variants (SNVs/InDels) found in the tumor biopsy, variants presented in the main text are highlighted. Columns are documented here: https://pcgr.readthedocs.io/en/v0.9.0rc/output.html#annotated-list-of-all-snvs-indels

Table S3 - Cancer-relevant genes screened for pathogenic germline variants with CPSR

Table S4 - Selected germline variants found in the blood sample (presented in the main text), as found through analysis with CPSR. Columns are documented here: https://cpsr.readthedocs.io/en/v0.6.0rc/output.html#tab-separated-values-tsv

Table S5 - Mutational signature contributions in the tumor biopsy. Columns are documented here: https://pcgr.readthedocs.io/en/v0.9.0rc/output.html#mutational-signature-contributions

Table S6 - Gene expression signatures interrogated with RNA-seq data

Table S7 - RNA fusion transcripts identified in the tumor biopsy. High-confident candidates highlighted. Columns are documented here: https://arriba.readthedocs.io/en/latest/output-files/

Table S8 - DNA primers used in PTEN deletion monitoring assay
